# A Low Dose of Nanoparticulate Silver Induces Mitochondrial Dysfunction and Autophagy in Adult Rat Brain

**DOI:** 10.1007/s12640-020-00239-4

**Published:** 2020-06-25

**Authors:** Joanna Skalska, Beata Dąbrowska-Bouta, Małgorzata Frontczak-Baniewicz, Grzegorz Sulkowski, Lidia Strużyńska

**Affiliations:** 1 Laboratory of Pathoneurochemistry, Department of Neurochemistry, Mossakowski Medical Research Centre, Polish Academy of Sciences, 5 A. Pawińskiego str., 02-106 Warsaw, Poland; 2 Electron Microscopy Platform, Mossakowski Medical Research Centre, Polish Academy of Sciences, 5 A. Pawińskiego str., 02-106 Warsaw, Poland

**Keywords:** Nanosilver, AgNPs, Autophagy, Myelin-like bodies, Mitochondria, Lysosomes, Rats

## Abstract

Extensive incorporation of silver nanoparticles (AgNPs) into many medical and consumer products has raised concerns about biosafety. Since nanosilver accumulates persistently in the central nervous system, it is important to assess its neurotoxic impacts. We investigated a model of prolonged exposure of adult rats to a low environmentally relevant dose of AgNPs (0.2 mg/kg b.w.). Ultrastructural analysis revealed pathological alterations in mitochondria such as swelling and cristolysis. Besides, elongated forms of mitochondria were present. Level of adenosine triphosphate was not altered after exposure, although a partial drop of mitochondrial membrane potential was noted. Induction of autophagy with only early autophagic forms was observed in AgNP-exposed rat brains as evidenced by ultrastructural markers. Increased expression of two protein markers of autophagy, beclin 1 and microtubule-associated proteins 1A/1B light chain 3B (MAP LC3-II), was observed, indicating induction of autophagy. Expression of lysosome-related Rab 7 protein and cathepsin B did not change, suggesting inhibition of physiological flux of autophagy. Our results show that exposure to a low, environmentally relevant dose of AgNPs leads to induction of autophagy in adult rat brain in response to partial mitochondrial dysfunction and to simultaneous interfering with an autophagic pathway. The cell compensates for the defective autophagy mechanism via development of enhanced mitochondrial biodynamic.

## Introduction

Extensive design, production, and use of many different types of metal nanoparticles (NPs), which possess unique physical, chemical, and biological properties, have occurred over the past few decades, resulting in development of applications in many different fields, including medicine (Klębowski et al. 2018). In particular, there has been tremendous interest in silver nanoparticles (AgNPs), which have recently been incorporated into many consumer products because of their strong antiseptic properties (Vance et al. 2015). Moreover, the biomedical-related potential of AgNPs has been assessed, and it has been proven that they can be used as antimicrobial agents, drug delivery carriers, detection and diagnosis platforms, medical device coatings (Burdușel et al. 2018; Melayie and Youngs 2005), and anticancer agents (Ullah Khan et al. 2018).

However, safety concerns have been raised over the extensive applications of NPs because their nanoscale properties makes them highly bioactive and potentially harmful to exposed organisms (Cronholm et al. 2013). In order to ensure the continued development of nanotechnology and the commercialization of products containing nanoparticles, it is necessary to identify all the health risks associated with using nanoscale materials and to determine their mechanisms of toxicity. Although AgNPs possess impressive advantages, which are clearly useful in biomedical applications, there is limited information regarding biological interactions and neurotoxic mechanisms of AgNPs, particularly after long-term exposure.

In vivo studies have demonstrated that AgNPs are translocated into the central nervous system (CNS) after oral administration (Loeschner et al. 2011). Nanogranules have been detected in various brain regions (cerebral cortex, hippocampus) after prolonged oral exposure (Skalska et al. 2015). Moreover, persistent accumulation of AgNPs in brain parenchyma has been revealed (Lee et al. 2013a), leading to alterations in locomotor activity (Yin et al. 2015) and impairment of spatial cognition (Liu et al. 2012) in rats. An array of in vitro and in vivo studies on mechanisms of AgNPs neurotoxicity have proven that exposure to AgNPs triggers overproduction of reactive oxygen species (ROS), which leads to oxidative stress. Excessive amounts of ROS can damage cellular structures (particularly mitochondria) and molecules such as DNA, proteins, and lipids, inducing inflammatory response, which leads to cell death in necrotic and/or apoptotic pathways (for reviews see: Skalska and Strużyńska 2015; Strużyńska and Skalska 2018). ROS production and oxidative stress are among the factors that induce both apoptosis and autophagy. A growing number of research reports have clarified the crosstalk occurring between the autophagy and apoptosis pathways, which may be triggered by common upstream signals. Cellular stress can induce an autophagy response, which represents a stress-adaptive mechanism against cell death or, alternatively, may cause apoptosis when it exceeds a certain threshold (which is lowered when autophagy is inhibited) (Maiuri et al. 2007).

Indeed, the significant role of autophagy in toxicity of nanomaterials, including AgNPs, has been emphasized (Stern et al. 2012). In vitro studies of AgNPs administration to the human liver cancer *cell line* HepG2 (Mishra et al. 2016) and the human neuroblastoma cell line SH-SY5Y (Li et al. 2019) revealed the presence of pro-autophagy proteins. Activation of autophagy in rat liver upon intraperitoneal administration of AgNPs has also been observed (Lee et al. 2013b). Moreover, it has been suggested that AgNPs may disrupt proper functioning of the autophagy process in human liver-derived hepatoma cells in vitro (Mishra et al. 2016).

Autophagy (mainly macroautophagy) is an evolutionarily conserved lysosomal degradation pathway, which plays a critical role in the homeostatic process of recycling of damaged proteins and organelles to help stressed cells survive under conditions such as nutrient deprivation, infection, oxidative stress, genotoxic stress, and hypoxia (Parzych and Klionsky 2014). Thus, autophagy is generally a life-preserving process whose upregulation is believed to be protective. After induction of autophagy, double-membrane structures (phagophores) are created in the cytoplasm which engulf damaged or obsolete proteins, organelles, and other cellular components or pathogens forming the autophagosome. Then the autophagosome fuses with a lysosome and forms an autolysosome, which degrades the encapsulated materials by lysosomal enzymes, generating components useful for cell growth and homeostasis (Xie and Klionsky 2007).

There is increasing evidence that autophagy is particularly important for maintaining homeostasis in the CNS where, apart from protecting against neurodegeneration, it regulates neurogenesis. A defective autophagy pathway has been linked to pathogenesis of several human disorders, including neurodegenerative diseases (Menzies et al. 2017; Shintani and Klionsky 2004). Moreover, regulation of this process has been suggested as a new therapeutic strategy (Rubinsztein et al. 2012; Giordano et al. 2014).

Since most of the investigations of AgNP-induced autophagy have been in vitro studies, in this study, we examined whether prolonged oral administration of AgNPs may induce and/or affect the process of autophagy in brain parenchyma of exposed adult rats. The study was designed to define the impact of a low environmentally relevant dose of AgNPs (0.2 mg/kg b.w.). Under such experimental conditions of exposure to AgNPs, ultrastructural and biochemical analysis of autophagic markers was performed (including analysis of expression of beclin 1, MAP LC3-II, Rab 7, and cathepsin B) together with estimations of mitochondrial condition (membrane potential, ATP level) and the expression of apoptosis-related proteins (Bcl-2 and Bax). The effects evoked by particulate and ionic forms of silver were compared according to our current knowledge that certain AgNP-induced toxic mechanisms are caused by silver ions released from the surfaces of nanoparticles (for a review see: Strużyńska and Skalska 2018).

## Materials and Methods

### Particulate and Ionic Silver

AgNPs (CAS No. 730785) and silver citrate (a source of silver ions) were purchased from Sigma-Aldrich Chemical Co. (St. Louis, MO, USA). The form of these AgNPs is defined by the manufacturer as a colloidal solution of nanoparticles (10 ± 4 nm in diameter) suspended in an aqueous citrate buffer in a concentration of 0.02 mg AgNPs/mL to provide long-term stability. According to the manufacturer, each batch of AgNPs is characterized to ensure a homogeneous product (monodisperse AgNPs free from agglomeration), as indicated by the following parameters: refractive index n20/D = 1.333, fluorescence—λ_em_ = 388 nm, and full width at half maximum value (FWHM) = 59 nm.

Additional characterization of these AgNPs was published in our previous study where we assessed the degree of dispersion and size distribution of AgNPs with a transmission electron microscope (JEM-1200EX, Jeol, Japan) equipped with a digital camera MORADA and iTEM 1233 software (Olympus Soft Imaging Solutions, GmbH, Germany) (Skalska et al. 2015).

### Animals and Experimental Design of Silver Exposure

Six-week-old male Wistar rats (total of 42), weighing initially 180–210 g, were used in the experimental procedures. Animals were obtained from the Animal House of the Mossakowski Medical Research Centre, Polish Academy of Sciences (Warsaw, Poland). They were housed two per cage in consistent environmental conditions (12 h light/dark cycle, 22 ± 1 °C, 55 ± 5% humidity). Purity of the air supplied to the rooms was provided by EU7 pre-filters and HEPA H13 filters with number of exchanges 20/h and laminar flow of air 0.5 m/s. Animals have free access to standard feed (rat breeding S8435-S022, SNIFF, Germany) and drinking water and were handled in accordance with the EU ethical guidelines for the care and usage of laboratory animals (Directive 2010/63/EU) and in compliance with the ARRIVE guidelines. The experimental procedures were approved by the local Experimental Animal Care and Use Committee.

Rats were randomly divided into three groups, each consisting of 14 animals and representing: (i) a negative control group exposed to saline, (ii) an AgNP-exposed group exposed to citrate-stabilized AgNPs at a dose of 0.2 mg/kg b.w., and (iii) a silver ion-exposed group (silver citrate administered as a source of Ag^+^) at a dose of 0.2 mg Ag^+^/kg b.w. Solutions were administered once daily via a gastric tube (AnimaLab, Poznań, Poland) for 14 consecutive days. Animals (*n* = 42) were either sacrificed by decapitation 24 h after administration of the final dose of examined solutions to obtain tissues for biochemical assays (*n* = 33) or were anesthetized and perfused for TEM analysis (*n* = 9).

### Determination of Silver with ICP-MS

After the last dose of AgNPs or silver citrate, rats were sacrificed and samples of tissues were prepared for analysis of silver concentration by ICP-MS (Elan 6100 DRC Sciex Perkin Elmer, Canada). Blood was collected from the neck after decapitation and centrifuged to obtain serum (1000×*g*, 4 °C, 10 min). Serum was then diluted with 1% Triton solution to analyze the concentration of silver.

The brains from three animals per group were isolated. The brain tissue was weighed, lyophilized, and digested with concentrated nitric acid. The concentration of silver was analyzed in the digested fluid. Quantification results were verified by the addition of 3 concentration levels of an internal standard (ICP Multielement Standard, TraceCERT(R), Sigma Aldrich).

### TEM Analysis of Brain Samples

After deep anesthesia with nembutal (80 mg/kg b.w.), the rats were perfused through the ascending aorta with 0.9% NaCl in 0.01 M sodium-potassium phosphate buffer (pH 7.4) and then with 2% paraformaldehyde and 2.5% glutaraldehyde in 0.1 M cacodylate buffer (pH 7.4). Brain samples were collected, fixed in the above ice-cold fixative solution, and post-fixed in 1% OsO_4_ solution. Then the samples were dehydrated in the ethanol gradient, embedded in epoxy resin (Epon 812) and cut into ultrathin sections, which were stained with 9% uranyl acetate and lead nitrate. Analysis of brain sections taken from cerebral cortex and hippocampus was performed by TEM (JEM-1200EX, Jeol, Japan) equipped with a digital camera MORADA and iTEM 1233 software (Olympus Soft Imaging Solutions, GmbH, Germany).

Additionally, another group of sections was not contrasting with metallic compounds (Pb, U) to avoid occurrence of osmium artifacts or lead precipitates. Omitting of this step allows visualization of the potential presence of silver in brain tissue, which is the only metal found in cellular structures during such tissue processing.

### Measurement of Mitochondrial Membrane Potential

The mitochondrial membrane potential was determined by measuring the uptake of the cationic carbocyanine dye JC-1 (5,5′,6,6′-tetrachloro-1,1′,3,3′-tetraethylbenz-imidazolcarbocyanine iodide) into the mitochondria. This dye undergoes changes in fluorescence properties after entering the mitochondrial matrix. In healthy cells with high membrane potential, JC-1 becomes concentrated in the matrix, where it forms bright red fluorescent complexes known as J-aggregates. However, any dissipation of the mitochondrial membrane potential inhibits the accumulation of the JC-1 dye in the mitochondria and triggers dispersion of the dye within the cytoplasm as green fluorescent monomers.

Freshly isolated brain tissue was rapidly homogenized in a cold buffer (5 mM HEPES, pH 7.4, containing 225 mM mannitol, 75 mM sucrose, 0.5 mM EGTA and 2 mg/mL albumin; proportion: 1 g of the sample per 10 mL of extraction buffer) to isolate a mitochondria-enriched fraction. After low-speed centrifugation of the homogenate (600×*g*, 5 min, 4 °C) and subsequent high-speed centrifugation of the obtained supernatant (11,000×*g*, 10 min, 4 °C), the final pellet containing a mitochondria-enriched fraction was suspended in a storage buffer (10 mM HEPES, pH 7.5, containing 225 mM sucrose, 2 mM K_2_HPO_4_, 1 mM ATP, 0.1 mM ADP, 5 mM sodium succinate and 1 mM DTT) in proportion: 40 μL of storage buffer per 100 mg sample.

After measuring the protein concentration (Lowry et al. 1951), the mitochondrial membrane potential of the inner mitochondrial membrane was determined using a commercial kit (Isolated Mitochondria Staining Kit, Sigma-Aldrich; St. Louis, MO, USA; CAS No. CS0760) according to the to the manufacturer’s instructions.

### Western Blot Analysis

The day after administration of the final dose, rats were sacrificed by decapitation. Brains were rapidly removed, washed in a cold phosphate buffer (pH 7.4), and homogenized in isolating medium containing 0.32 M sucrose, 1 mM ethylenediaminetetraacetic acid (EDTA), and protease inhibitor cocktail. The protein concentration of brain homogenates was determined by the method of Lowry et al. (1951). A standard curve was generated using bovine albumin.

Equal amounts of proteins (50 μg) were subjected to sodium dodecyl sulfate polyacrylamide gel electrophoresis (SDS-PAGE) and then transferred onto nitrocellulose membranes 0.45 μm Hybond-Extra C (Amersham, U.K.). The membranes were incubated overnight with the primary antibodies: mouse anti-beclin 1 (Santa Cruz Biotechnology, Dallas, USA; 1:1000), mouse anti-MAP LC3-II (Santa Cruz Biotechnology, Dallas, USA; 1:1000), mouse anti-Rab7 (Sigma-Aldrich; St. Louis, MO, USA; 1:1000), mouse anti-cathepsin B (Sigma-Aldrich; St. Louis, MO, USA; 1:250), mouse anti-Bcl-2 (Sigma-Aldrich; St. Louis, MO, USA; 1:500), and rabbit anti-Bax (Sigma-Aldrich; St. Louis, MO, USA; 1:1000). Then the membranes were incubated with secondary anti-rabbit or anti-mouse (Sigma-Aldrich; St. Louis, MO, USA; 1:10000) antibodies conjugated with HRP for 30 min. Mouse monoclonal anti-β-actin antibody (MP Biomedicals, Warsaw, Poland; 1:500) was used as an internal standard. Bands were visualized on Hyperfilm ECL using the chemiluminescence ECL kit (Amersham, U.K.). The films were scanned and quantified using ImageJ software.

### Measurement of ATP Levels

ATP in the brain was measured using an ATP assay kit (Abcam, Cambridge, UK) according to the manufacturer’s instructions. Briefly, 10 mg of brain tissue was homogenized in 2 N ice-cold perchloric acid (PCA), and the supernatant was diluted with ATP assay buffer. The samples were neutralized and deproteinized with 2 M potassium hydroxide (KOH) and loaded in triplicate into a microplate reader. The optical density (OD) was measured at 570 nm using a FLUOstar Omega spectrophotometer (Ortenberg, Germany). Values of ATP levels were calculated relative to the standard curve.

### Statistical Analysis

The results from protein expression and mitochondrial membrane potential analyses are expressed as mean ± SD from the experiments performed using the number of animals indicated in the figure legend. Differences between groups were compared using the one-way analysis of variance (ANOVA) followed by Dunnett’s multiple comparison posttests. *P* < 0.05 was considered significant. All analyses were performed using GraphPad Prism Software, version 6.0 (San Diego, CA, USA).

## Results

Commercially available citrate-stabilized AgNPs (mean diameter 10 ± 4 nm) were administered to the animals at a dose of 0.2 mg/kg b.w. According to the manufacturer, the AgNPs do not agglomerate in citrate solution and are retained in the form of distinct spherical nanoparticles of homogenous size. Our current and previous TEM studies (Skalska et al. 2015) confirmed the dispersed state of the AgNPs and revealed that only 5% of them are larger or smaller in diameter than 10 nm.

**Fig. 1:**
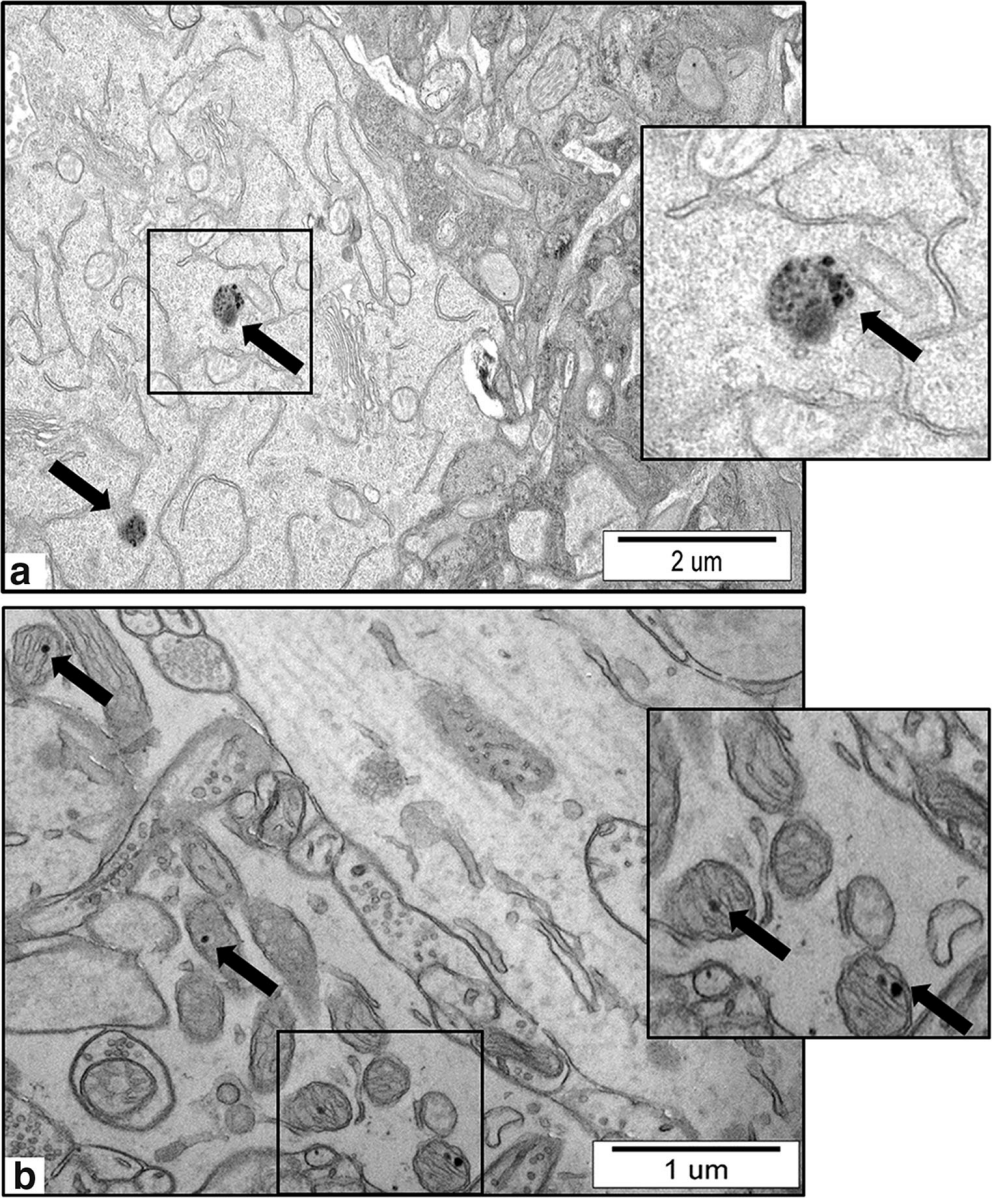
Representative TEM micrographs of cerebral sections from AgNP-exposed animals. **a** Nanogranules inside the lysosomes of neurons (arrows); **b** nanogranules in neuronal mitochondria (arrows). Ultrathin sections were not stained with uranyl acetate and lead citrate to visualize the presence of AgNPs, which may be observed as round dark nanosized granules. Insets were magnified 3–4×. Images are typical for each of 3 examined animals per group

Exposure either to AgNPs or silver citrate did not influence the body weight or the general appearance of the animals. The concentration of Ag in blood of the rats was found to be in the same range in both of exposed groups and to exceed significantly the control level (0.9 ± 0.2 μg/L) reaching a value of 12 ± 1.8 μg/L (*P* < 0.01 vs. control). However, we found that the concentration of silver in brain homogenates was below the detection limit for ICP-MS (0.241 μg/kg in solid tissue). Therefore, we demonstrated the presence of AgNPs in brain tissue indirectly, using a modified TEM method as described in “Materials and Methods.” We observed nanosized granules within a diameter range similar to the diameter range of administered AgNPs exclusively in brain of AgNP-exposed rats. These nanosized granules were located *i.a.* inside neuronal lysosomes and mitochondria (Fig. 1 a and b).

### The Influence of a Low Dose of AgNPs on Mitochondrial Status in Brain of Exposed Rats: Ultrastructural and Biochemical Alterations

In this study, we investigated a low dose of AgNPs (0.2 mg AgNPs/kg b.w. /day), which is believed to be relatively close to a base level of environmental contamination. This level was calculated based on the theoretical value, the predictable no-effect concentration (PNEC), which is in the range of 0.04–0.1 mg/L for water compartments according to the literature data (Hull and Bowman 2014).

As mentioned above, in the applied model of oral exposure to a low dose of AgNPs, we were not able to detect silver in brain homogenates of exposed rats using ICP-MS but revealed the presence of nanoparticles using a modified TEM analysis (see “Materials and Methods”).

**Fig. 2:**
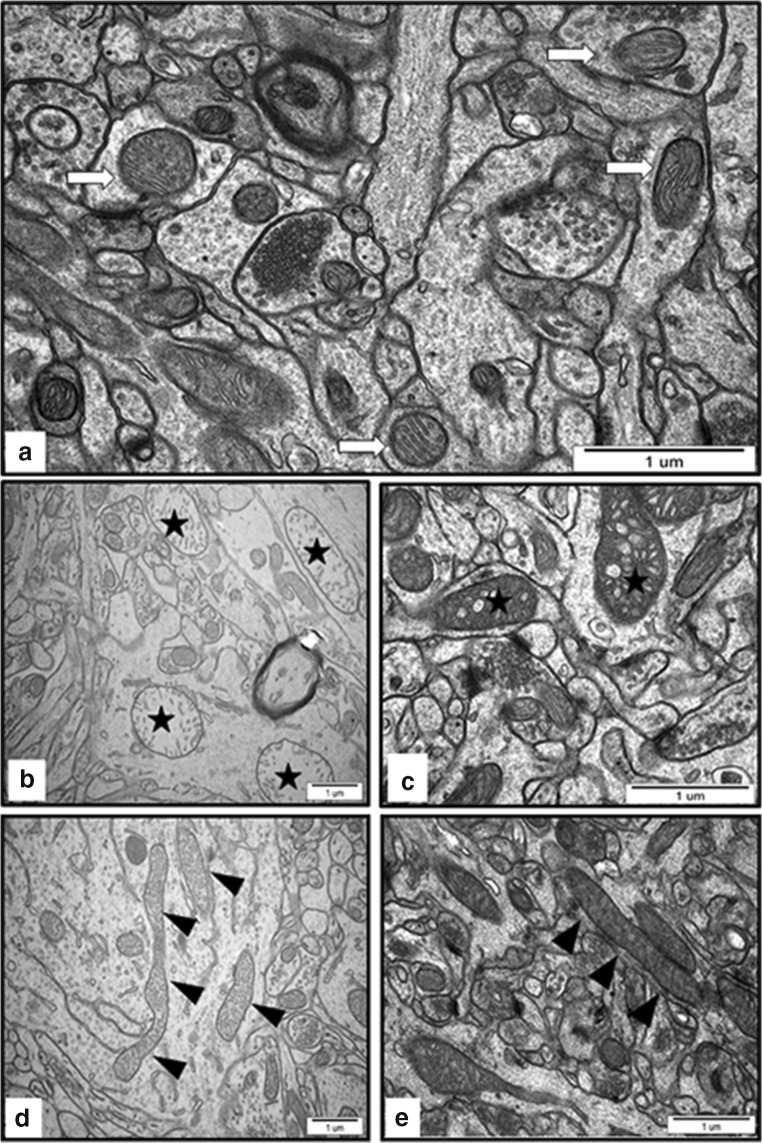
Representative TEM images from brain tissue of control (**a**) and AgNP-exposed (**b**–**e**) animals. **a** Normal structure of neuropil with normal appearance of mitochondria (white arrows) and well-defined structure of synapses. **b**, **c** An overview of neuropil with swollen mitochondria with fragmentation of the cristae (asterisks) in hippocampus of AgNP-exposed animals. **d**, **e** Elongated mitochondria in cerebral cortex of AgNP-exposed animals (arrowheads). Images are typical for each of 3 examined animals per group

In specimens obtained from brains of control animals (treated with saline), we observed a proper structure of neuropil with normal appearance of mitochondria and other cellular structures (Fig. 2a). TEM images of brains of AgNP-exposed rats show ultrastructural alterations in mitochondria. Pathological changes in neuronal mitochondria, often swollen and/or with disintegrated cristae (Fig. 2 b and c), were primarily observed in neurons located in the vicinity of leaky microvessels. Another feature of mitochondria, frequently observable in cerebral cortex, was their elongated shape (Figs. 2d, e and 4b), which is a known characteristic of autophagy.

It is notable that the profile of ultrastructural changes was found to differ between the two silver-treated groups. Elongated mitochondria were particularly evident in neurons of AgNP-exposed rats, whereas damaged and swollen mitochondria were observed in both silver-exposed groups, although more frequently in the AgNP- than in the Ag citrate–treated animals.

**Fig. 3:**
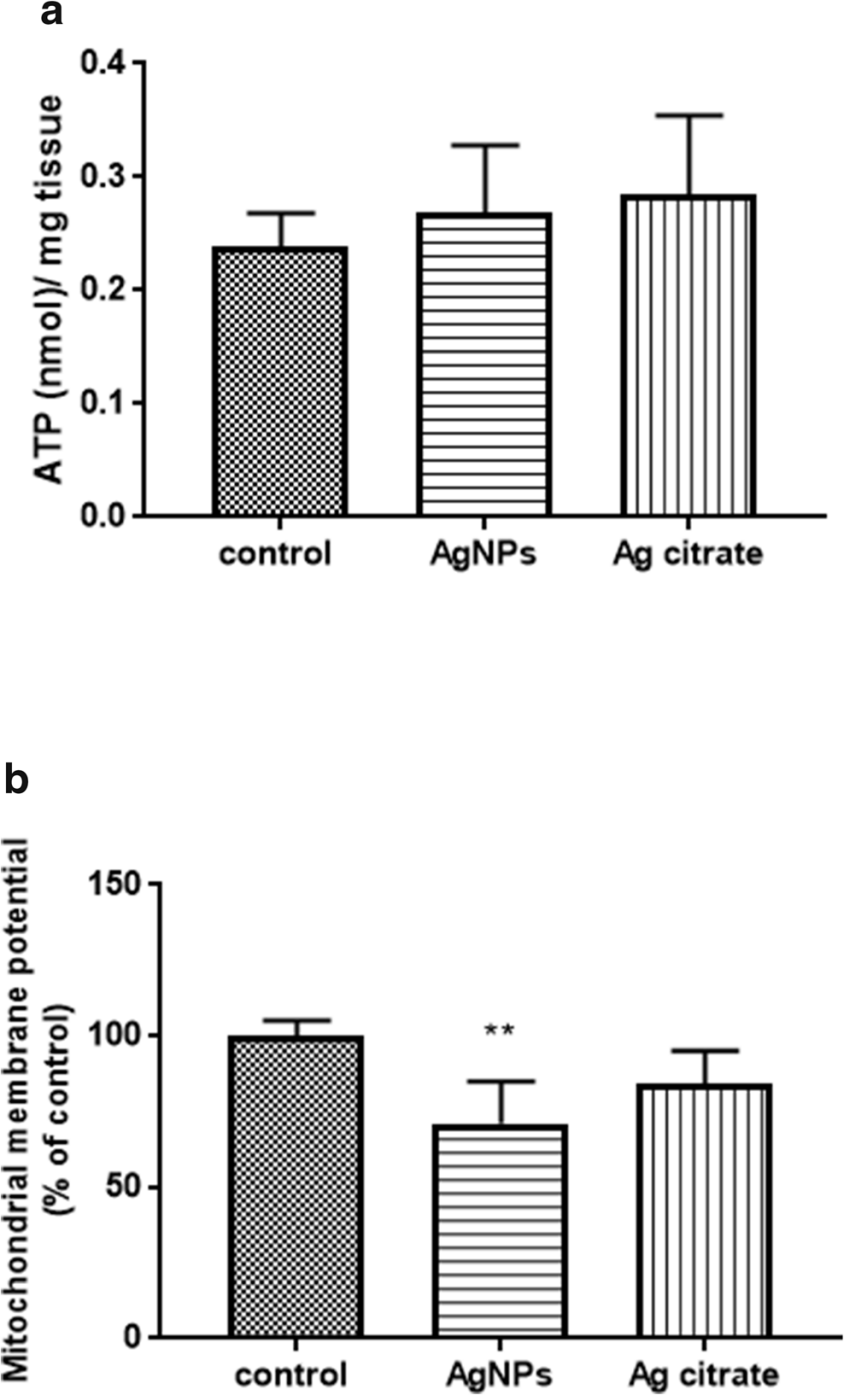
The level of ATP (nmol/mg tissue) (**a**) and mitochondrial membrane potential (**b**) in brain homogenates obtained from control (saline), silver nanoparticles (AgNPs), and silver citrate-exposed rats, determined by measuring the uptake of the dye JC-1. The results are the means ± SD from 4 distinct animals per group expressed as a percentage of control. **P < 0.01 indicates a significant difference vs. the control group (one-way ANOVA with post hoc Dunnett’s test)

Parallel to the ultrastructural changes in mitochondria, we observed dysfunction of these organelles. To investigate the effect of AgNPs and Ag^+^ on mitochondrial function, the mitochondrial membrane potential was first evaluated using the fluorescent dye JC-1. An electrochemical proton gradient exists across the inner mitochondrial membrane, which generates a voltage gradient (membrane potential; ΔѰm), which is negative inside and positive outside. We observed a significant decrease in ΔѰm exclusively in brain of rats from the AgNP-exposed group, by about 29% relative to the control group (*P* < 0.01 vs. untreated control) (Fig. 3b). However, despite the drop of ΔѰm, there was no significant change in ATP concentration in brain homogenates between the control group and the two study groups (Fig. 3a).

### Ultrastructural and Biochemical Markers of Autophagy in Brain of AgNP-Exposed Rats

The presence of nanosized granules inside lysosomes and mitochondria, an altered neuropil ultrastructure, and a decrease in mitochondrial membrane potential were observed in the AgNP-exposed group. This motivated us to further characterize the alterations in the expression of selected markers of autophagy and of lysosomal functions.

**Fig. 4:**
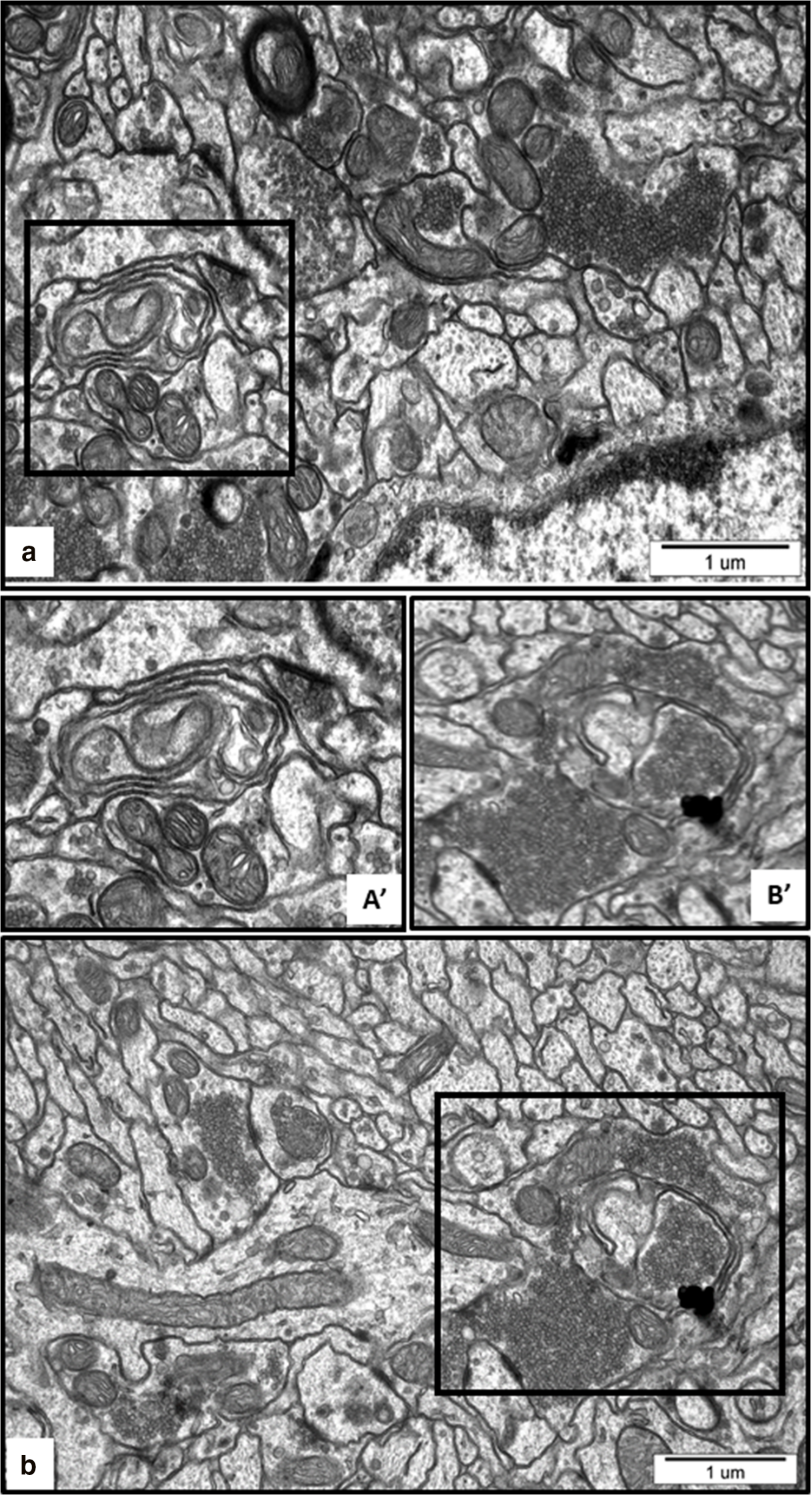
Representative TEM images from cerebral cortex of AgNP-exposed animals showing early stages of autophagosomes formation. Fragments of membranes surrounding remnants of cellular structures are indicated (a, A′) as well as clusters of synaptic vesicles liberated from the damaged synapse (b, B′) and elongated mitochondria (b). The insets A', B' are magnified 2×. Images are typical for each of 3 examined animals per group

**Fig. 5:**
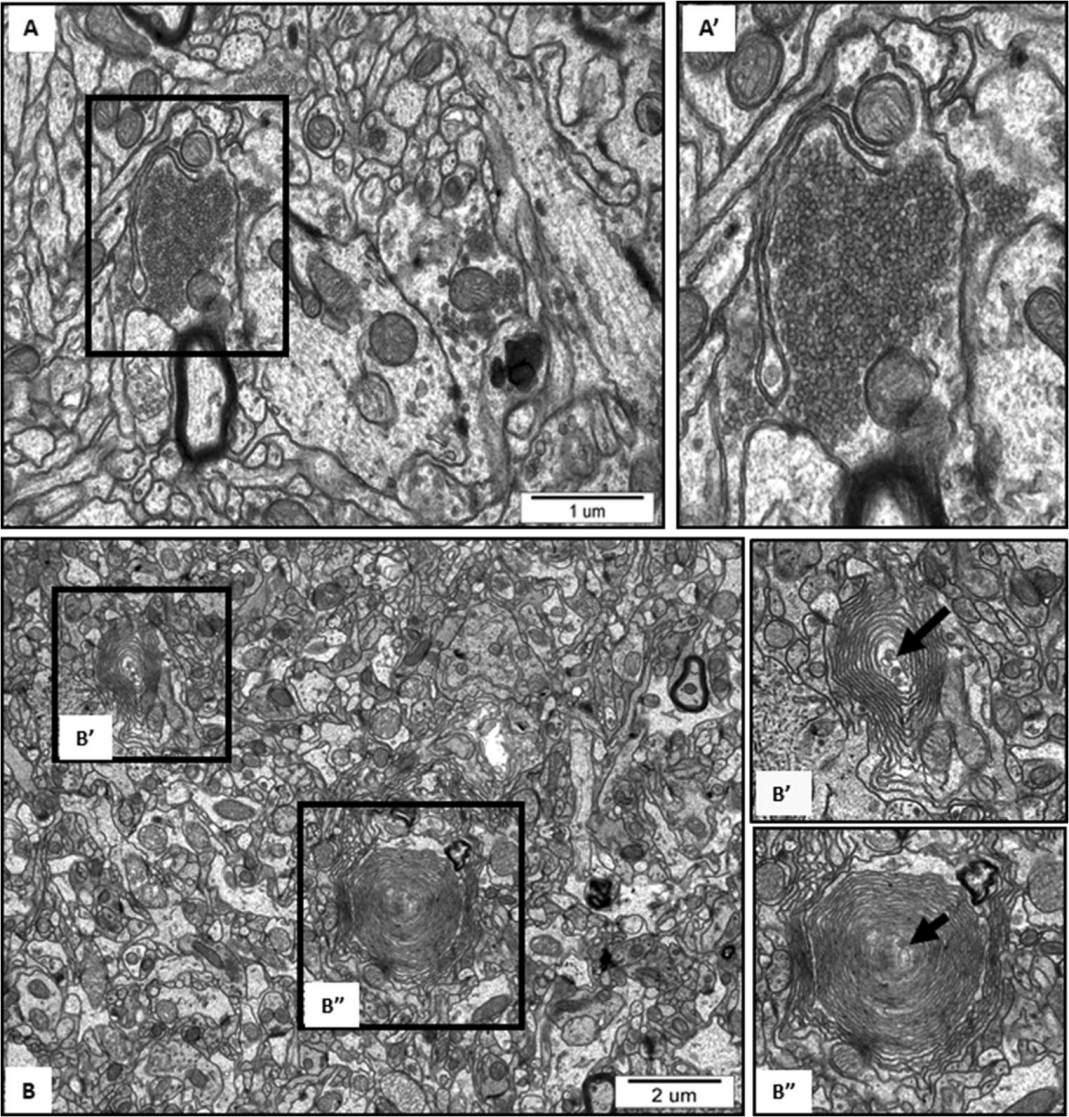
Representative TEM images from hippocampus of AgNP-exposed animals showing the initial stages of formation of autophagosomes. (A) Fragment of membrane surrounding the cluster of synaptic vesicles liberated from the damaged synapse and loosely located in the neuropil. (B) Myelin-like bodies—concentrically layered structures derived from fragmented membranes, bearing remnants of damaged organelle inside (B′, B″; arrows). The insets A', B', B'' are magnified 2–3×. Images are typical for each of 3 examined animals per group

Ultrastructural analysis revealed the cellular structures related to the process of autophagy in brains of AgNP-exposed rats such as isolation membranes surrounding damaged organelles, which represent the initial stages of formation of autophagosomes (Figs. 4 and 5). Membranous whorls create concentrically layered multilaminated structures, which are known as myelin-like bodies. These autophagy structures contain remnants of disturbed mitochondria and synaptic vesicles (Figs. 4 and 5). As we have previously reported, these observations indicate the occurrence of significant synaptic degeneration under exposure to AgNPs (Skalska et al. 2015). Importantly, autophagy structures such as myelin-like bodies were observed particularly in AgNP-exposed rats, most frequently (but not exclusively) in hippocampus.

**Fig. 6:**
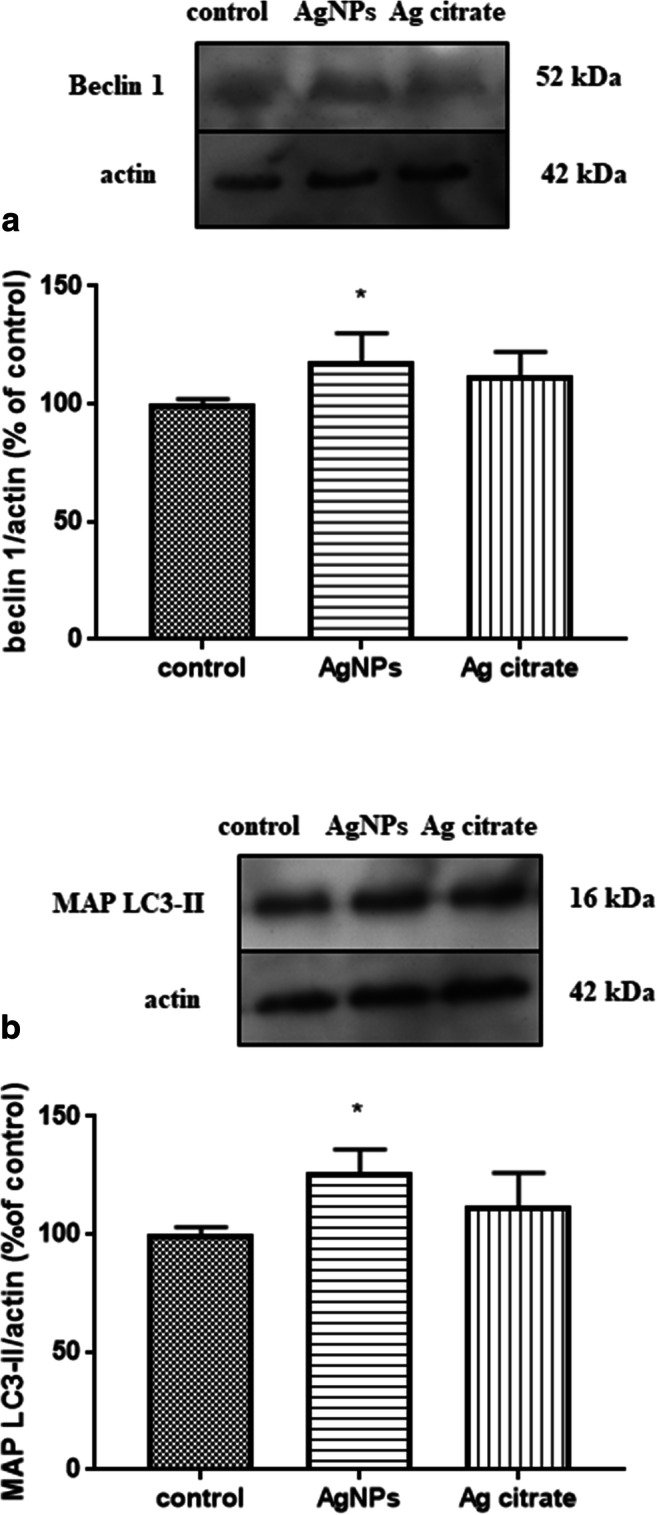
Expression of autophagy-related proteins involved in autophagosome formation in brain homogenates obtained from rats exposed to control (saline), silver nanoparticles (AgNPs), and silver citrate; Representative immunoblots for protein levels of beclin 1 (**a**) and MAP LC3-II (**b**). The graphs illustrate the results of densitometric measurements of 4 independent immunoblots performed using 4 distinct animals, expressed as a percentage of control. The relative protein density was measured against β-actin as an internal standard. The values represent the means ± SD, *P < 0.05 vs. control (one-way ANOVA with post hoc Dunnett’s test)

We further elected to characterize the expression of proteins involved in the autophagosome formation (beclin 1 and MAP LC3-II) and in lysosomal functions (cathepsin B and Rab7). We observed changes in the expression of proteins involved in formation of autophagosomes after exposure to AgNPs. The relative levels of beclin 1 and MAP LC3-II protein in brain homogenates were found to be elevated significantly in the AgNP-exposed group by about 16% and 27%, respectively (*P* < 0.05 vs. untreated control). The expression levels of these proteins were not found to be significantly altered relative to controls in the group treated with silver citrate (Fig. 6 a and b).

**Fig. 7:**
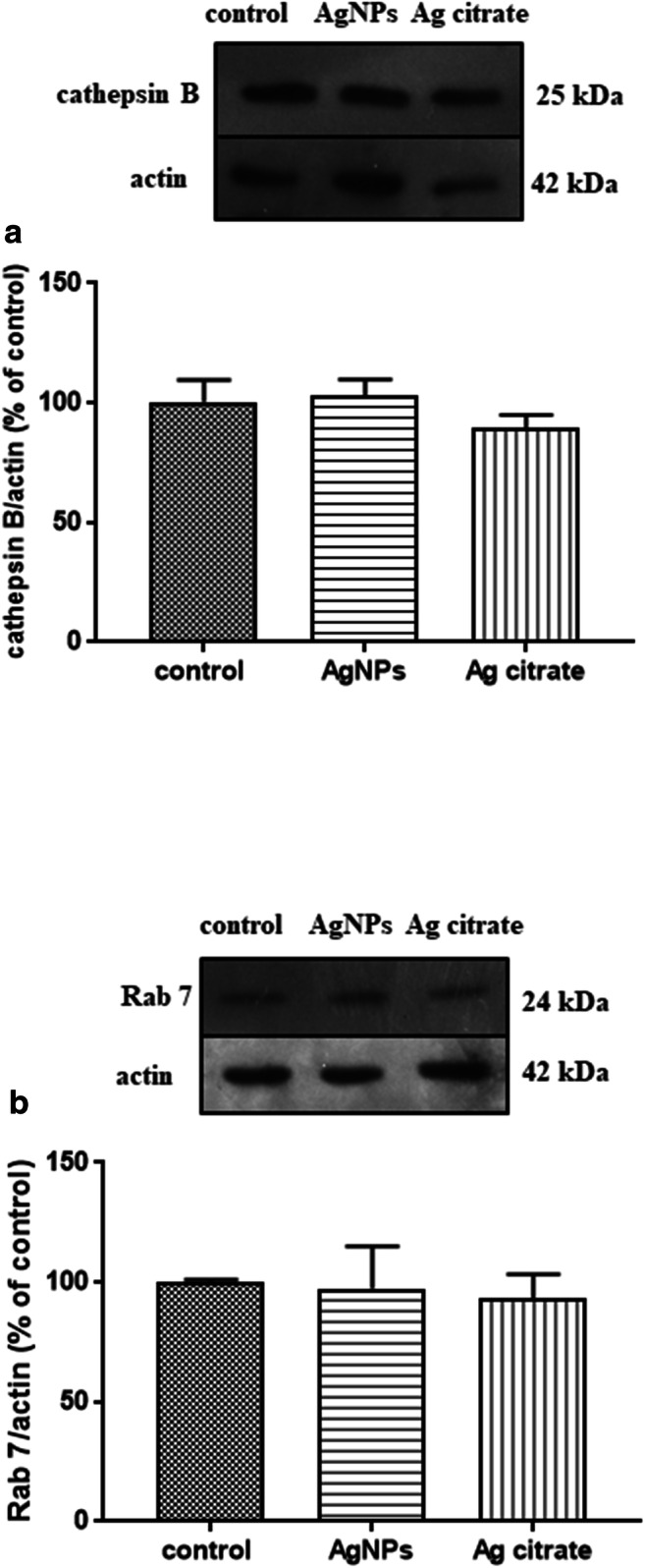
Expression of proteins involved in lysosomal functions during autophagy in brain homogenates obtained from rats exposed to control (saline), silver nanoparticles (AgNPs), and silver citrate; representative immunoblots for protein levels of cathepsin B (**a**) and Rab 7 (**b**). The graphs illustrate the results of densitometric measurements of 4 independent immunoblots performed using 4 distinct animals, expressed as a percentage of control. The relative protein density was measured against β-actin as an internal standard. The values represent the means ± SD; *P* > 0.05 was considered not significantly different vs. control group (one-way ANOVA with post hoc Dunnett’s test)

We did not identify statistically significant changes in the expression of cathepsin B and Rab7 protein in the two study groups (Fig. 7 a and b).

**Fig. 8:**
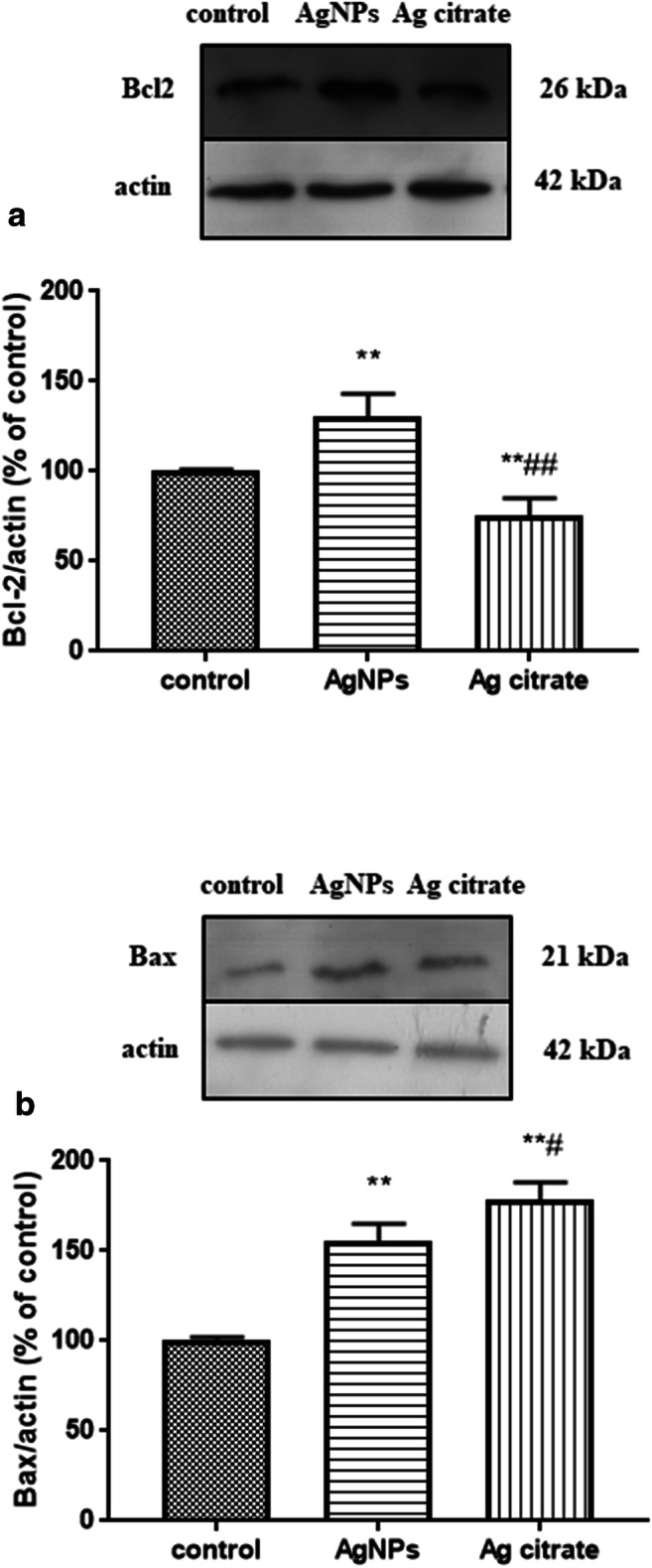
Expression of apoptosis-related proteins in brain homogenates obtained from rats exposed to control (saline), silver nanoparticles (AgNPs), and silver citrate; representative immunoblots for protein levels of Bcl-2 (**a**) and Bax (**b**). The graphs illustrate the results of densitometric measurements of 4 independent immunoblots performed using 4 distinct animals, expressed as a percentage of control. The relative protein density was measured against β-actin as an internal standard. The values represent the means ± SD, **P < 0.01; vs. control and ^#^*P* < 0.05; ^##^*P* < 0.01 vs. AgNPs (one-way ANOVA with post hoc Dunnett’s test)

Since we observed depolarization of the mitochondrial membrane potential after exposure to AgNPs (a phenomenon known as an early event in mitochondria-mediated apoptosis), we intended to measure the expression of Bcl-2 and Bax proteins, which are known as anti-apoptotic and pro-apoptotic proteins, respectively. Although ultrastructural alterations typical for apoptosis were not visible in neurons of the silver-treated rat brains, we observed a significant increase in the relative levels of apoptosis-related proteins. Pro-apoptotic Bcl-2 and anti-apoptotic Bax were increased in the AgNP-exposed group relative to the control group by about 30% and 55%, respectively (*P* < 0.01 vs. untreated control). In the Ag^+^-exposed group, the relative level of Bcl-2 was found to be remarkably lower (by about 25%) relative to the control value. The relative protein level of Bax protein in this group was significantly elevated by about 77% relative to the control value (Fig. 8). The Bax/Bcl-2 ratio was 1.15 and 2.43 for AgNP- and silver citrate-exposed groups, respectively.

## Discussion

### The Impact of a Low Dose of AgNPs on Neuronal Mitochondria

Significant quantities of toxins can accumulate in mitochondria and/or cause selective damage to these cellular structures. The first objective of the study was to investigate whether AgNPs administered in a low dose can reach the brain and induce pathological processes in mitochondria. Although we were not able to detect low concentrations of AgNPs in the brain by ICP-MS, we identified silver nano-granules in mitochondria and lysosomes while analyzing non-contrasted brain specimens by TEM (Fig. 1 a and b). We also observed that the silver nano-granules accumulating in mitochondria induce pathological alterations such as swelling and damage of cristae (cristolysis) (Fig. 2 a and b). Mitochondria are cellular osmometers, which react to traumatic or toxic insults by swelling and transforming into structureless mitochondrial vacuoles in severe cases. Swelling and cristolysis are suggestive of dysfunctionality of these intracellular organelles. Apart from the ultrastructural abnormalities, we identified a significant decrease in mitochondrial membrane potential ΔѰm measured in brain homogenates of rats treated with AgNPs but not in rats treated with silver citrate (Fig. 3b). ΔѰm is a basic index of mitochondrial activity and condition because damaged or functionally impaired mitochondria lack membrane potential. Depolarization of mitochondrial membranes may trigger the process of mitochondrial autophagy (mitophagy) (Eiyama and Okamoto 2015) and is also known to be an early event in apoptosis. Our results identifying AgNP-induced dysfunction of mitochondria are in agreement with previous reports showing that neuronal mitochondria are targeted by AgNPs, which may interact with these structures and perturb their function (Xu et al. 2013; Zieminska et al. 2014). In addition to swollen mitochondria, we identified elongated mitochondria in brain tissue of AgNP-exposed rats (Fig. 2d, e). Elongation is a result of enhanced fusion activity and has been reported as a typical response of mitochondria to stress conditions (Gomes et al. 2011).

Mitochondria are the energy centers of the cell, which generate ATP to provide energy for all cellular processes. Although partial depolarization of mitochondrial membranes was observed to occur in AgNP-exposed rats, surprisingly we did not observe a reduction in ATP levels (Fig. 3a). However, as mentioned above, we identified elongated mitochondria in the disturbed tissues. Mitochondrial elongation has been described as a process occurring during autophagy, which is critical to sustain cell viability and to determine the fate of the cell. Upon fusion, longer mitochondria are protected from degradation while being simultaneously more efficient in energy production (Gomes et al. 2011). The process of elongation increases the number of cristae per surface, which optimizes ATP production and results in an increased cell viability. Presumably, there is a balance between disturbed and elongated mitochondria, which sustains the normal ATP levels measured in brain tissue of AgNP-exposed rats.

However, concomitantly with production of ATP, mitochondria generate reactive oxygen species during oxidative phosphorylation. Dysfunctional mitochondria may further increase production of free radicals thereby inducing oxidative stress. Indeed, the vast majority of in vitro and in vivo data provide strong support for the concept that oxidative imbalance and subsequent oxidative stress are among the most important pathological events induced by AgNPs (for reviews see: McShan et al. 2014; Skalska and Strużyńska 2015). In accordance with these reports are our own studies performed previously under the same conditions that demonstrate increased production of ROS and peroxidation of lipids, as well as lowering of the reduced-to-oxidized glutathione ratio (GSH/GSSG) in brain of rats treated with a low dose (0.2 mg/kg b.w.) of AgNPs (Skalska et al. 2016). It has been suggested that the autophagy pathway may serve as an antioxidant mechanism, which clears proteins and/or organelles that are damaged by the excessive ROS produced during oxidative stress. By breaking down and recycling macromolecules, autophagy contributes to improvement of the cellular energy state (Giordano et al. 2014).

### Autophagy Is Induced in Brain of Rats Subjected to a Low Dose of AgNPs but Not in Rats Treated with Silver Citrate

Concomitant presence of disturbed mitochondria without reduction of ATP levels in AgNP-exposed rats is intriguing. Therefore, the next objective, based on the initial results indicating mitochondrial alterations, was to search for evidence of induction of autophagy. Autophagy, which normally occurs at a basal level in the cell, is enhanced by different stressors, thus being generally regarded as a manifestation of cell injury.

We found several prerequisites for induction of autophagy under exposure to a low dose of AgNPs which were (i) depolarization of mitochondrial membranes which may trigger the process of mitochondrial autophagy (mitophagy) (Eiyama and Okamoto 2015); (ii) elongation of mitochondria via a process known to occur in response to autophagic stimuli in order to protect these structures from autophagic degradation (Gomes et al. 2011); (iii) the presence of disrupted/dysfunctional mitochondria, which should be removed via the autophagic process of “cellular cleaning”; (iv) sustained levels of ATP despite partial depolarization of mitochondria, which may be a result of autophagy improving the cellular energy state (Giordano et al. 2014); and (v) AgNP-induced oxidative stress (Skalska et al. 2016), which may contribute to amplification of autophagy.

First, we identified ultrastructural characteristics confirming the induction of autophagy in AgNP-exposed rat brain tissues. The initial autophagic structures are formed in membranes originating from the endoplasmic reticulum or other disturbed cellular structures. Autophagosome-forming sequestering membranes are referred to as phagophores. Such structures are seen in Fig. 4 and Fig. 5(A). As previously reported (Cheville 2009), a marked whorling of membranes, which often surround mitochondria and lipid/protein globules, is a common characteristic of autophagy. The presence of synaptic vesicle clusters (Fig. 4(B)) or mitochondrial remnants (Fig. 4(A)) within whorled membranes is presumed to be a manifestation of this phenomenon. In case of excessive breakdown of membranes accompanied by inadequate debris degradation (overload of ubiquitination or exhaustion of lysosomal enzymes), synaptic vesicle clusters accumulate in the cytoplasm in the form of concentric lamellar arrangements (Cheville 2009), which are known as myelin-like bodies. It has been suggested that myelin-like bodies develop during autophagy (Kelekar 2005). We observed such structures bearing remnants of damaged organelles (likely mitochondria and synaptic vesicles) exclusively in AgNP-exposed rat brain tissue (Fig. 4(B)). Similar formations were observed in an experimental model of excitotoxicity-induced axonopathy (Saggu et al. 2010) and during retinal neurodegeneration (Schuettauf et al. 2004).

The autophagy pathway is regarded as the main mechanism for elimination of aberrant cell components and is persistently activated under different stress conditions, including toxic brain injury, and it is believed to be a central component of the integrated stress response (Kroemer et al. 2010). As we reported previously, exposure to AgNPs leads to production of reactive oxygen species (ROS), oxidative stress (Skalska et al. 2016), and disrupted function and structure of mitochondria (Zieminska et al. 2014). Therefore, induction of autophagy may represent a cellular mechanism that is activated to remove oxidatively damaged proteins and/or organelles. It has been hypothesized that activation of this process reduces spreading of oxidative stress in neuronal tissue. Thus, induction of autophagy can be regarded as a specific antioxidant mechanism (Giordano et al. 2014). Autophagy exerts a protective effect in neuronal injury and, as such, is particularly important for maintenance of axon terminals and in protection against axonal degeneration (Cheville 2009; Menzies et al. 2017). Indeed, we previously provided biochemical and ultrastructural evidence that synaptic degeneration is a characteristic feature observed under exposure to AgNPs (Skalska et al. 2015). In light of these findings, induction of autophagy highlights an early stage of axonal dysfunction.

**Fig. 9:**
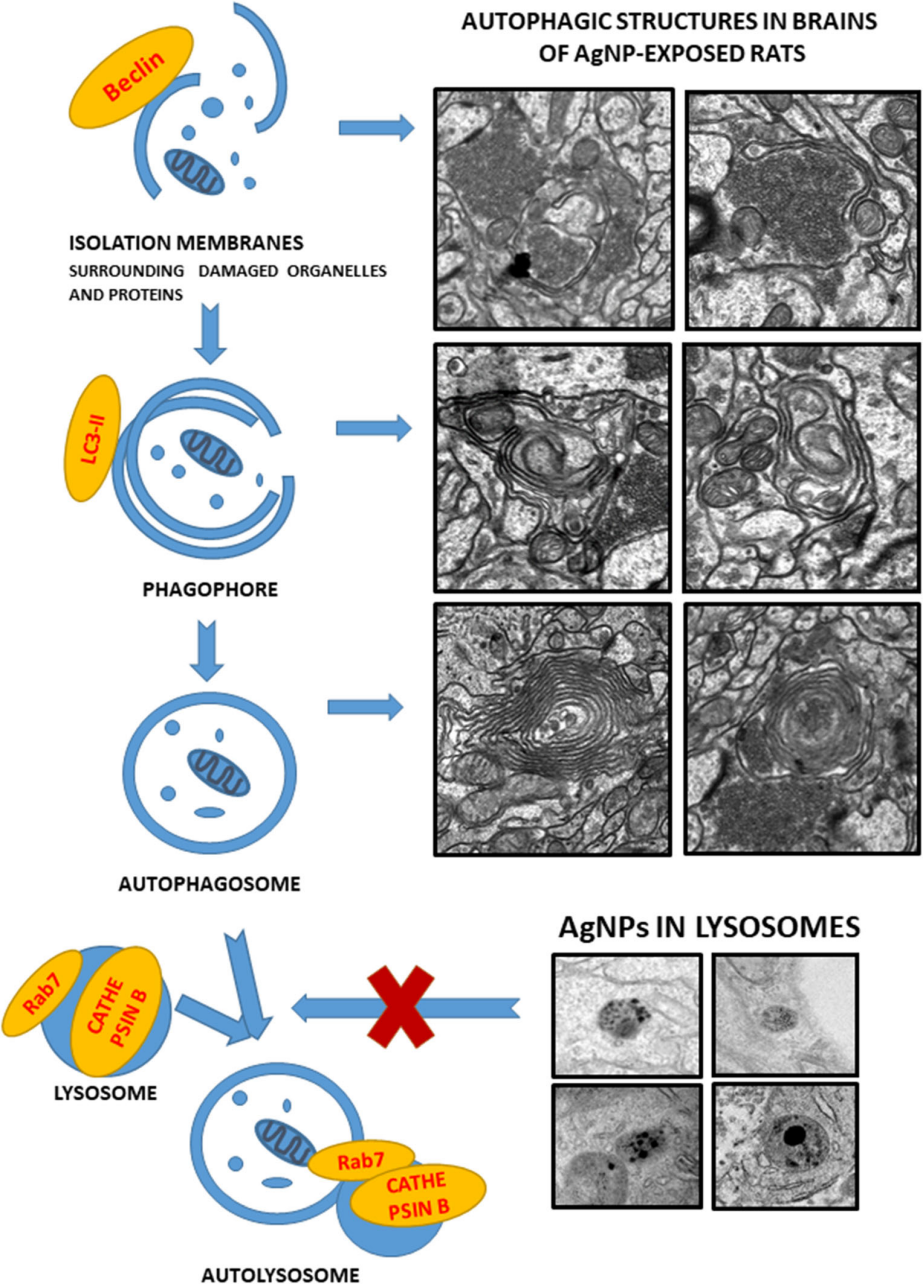
A schematic course of autophagy (left) and the proposed impact of AgNPs on different stages of this process (right) based on the results of our study

The onset of autophagy processes caused by exposure to AgNPs was also confirmed biochemically by observation of increased relative expression of proteins related to the early steps of the autophagy pathway such as beclin 1 and LC3-II, which are tightly associated with autophagosomal membranes (Menzies et al. 2017) and widely used for quantification of autophagy activity (Kabeya et al. 2000). We investigated expression levels of two additional proteins of lysosome origin. Rab7 has been shown to be associated with late endocytic structures, mainly lysosomes (Bucci et al. 2000), whereas cathepsin B is a cysteine protease of the endolysosomal compartment. Expression levels of these two proteins are not increased in AgNP-exposed rats. Rab7 has been suggested to have regulatory functions being essential, *i.a.* for the autophagosome-lysosome fusion and for subsequent degradation of cellular debris (Nakamura and Yoshimori 2017). Upon induction of autophagy, the expression of this protein should increase after fusion of autophagosomes with lysosomes (Gutierrez et al. 2004). This did not occur in our study, suggesting that under exposure to AgNPs, this step of the autophagy process may be blocked (Fig. 9).

Indeed, our TEM analysis revealed the presence of early autophagy structures but not late autophagy structures, as well as aberrant autophagosomal membranes, which are assumed to be a phenotypic hallmark of a disturbed autophagy process (Mao et al. 2016). Furthermore, AgNP granules are visible in neuronal lysosomes and mitochondria of rat brain tissue exposed to AgNPs. This observation is consistent with existing data indicating accumulation of AgNPs in the cellular lysosomal compartment and liberation of silver ions from the surface of AgNPs in the acidic environment of lysosomes, which is favorable for releasing ions from endocytosed AgNPs (Setyawati et al. 2014). AgNPs or Ag^+^ liberated from the surfaces of AgNPs may contribute to lysosomal dysfunction. As proposed by Mao et al. (2016), overloading of lysosomes with nanoparticles may lead to their impairment through lysosomal membrane permeabilization, inactivation of lysosomal enzymes, or alkalinization of their internal environment. Undoubtedly, AgNPs are capable of inducing autophagy in rat brain, even under condition of a low-dose exposure, as indicated by the results of our study. However, the question arises whether the autophagy pathway operates properly, thereby providing a protective role as suggested by Li et al. (2019), or whether it is inhibited. Our results are consistent with earlier in vitro studies which suggested that AgNPs can block autophagy flux by interfering with lysosomal functions (Xu et al. 2015) and destabilizing the autophagy-lysosomal system (Mishra et al. 2016). Our observations are also in line with previous reports indicating that other types of nanoparticles, such as graphene oxide quantum dots (Ji et al. 2016) and silica NPs (Wang et al. 2017), may block autophagy by inhibiting lysosome proteolytic activity in vitro*.* Moreover, based on our current and previous results, we found that there is a critical relationship between mitochondrial dysfunction, oxidative stress, and autophagy. In this context, AgNPs act in a manner similar to other types of NPs (for a review see: Li and Ju 2018).

It should be highlighted that a dysfunctional mechanism of autophagy may be detrimental to the CNS. Impairment of lysosomal function as well as autophagy flux has been shown to contribute to neuronal degeneration and to pathogenesis of a number of neuro-degenerative diseases (Menzies et al. 2017).

Based on the concept of autophagy/apoptosis antagonism (Kroemer et al. 2010) and our observation that AgNPs induce depolarization of mitochondrial membranes in an early event in the apoptotic pathway, we assessed the expression of pro- and anti-apoptotic proteins. The integrity of mitochondria is regulated by proteins from the Bcl-2 superfamily, among which the pro-apoptotic proteins Bax and Bak are essential for inducing apoptosis, whereas Bcl-2 protein inhibits mitochondria permeabilization and blocks the apoptotic pathway by interacting with Bax and Bak (Li et al. 2016). The profile of changes in expression of Bax and Bcl-2 proteins in the current study did not suggest the induction of apoptosis in AgNP-exposed animals. A significant increase of the Bax/Bcl-2 ratio, which is considered a better prognostic criterion of cellular apoptotic potential than expression of individual proteins, was observed in rats treated with silver citrate but not in AgNP-exposed rats (Bax/Bcl-2 = 2.43 vs. 1.15, respectively). However, ultrastructural analysis did not yield evidence for apoptosis in both of the study groups. Our results conflict with those reported by Lee et al. (2013b) who found that autophagy dysfunction leads to induction of apoptosis in liver of rats treated with high doses of AgNPs (500 mg/kg b.w.). This may indicate that induction of autophagy occurs even at low concentrations of AgNPs, whereas apoptosis is rather dose-related and occurs when the threshold of the protective autophagy response is exceeded.

In conclusion, we provide biochemical and ultrastructural evidence that exposure to a low, environmentally relevant dose of AgNPs induces autophagy in brain tissue of rats in response to mitochondrial dysfunction. Given the vital role of autophagy in brain homeostasis, it should be regarded as a protective mechanism whose role is to remove dysfunctional mitochondria and maintain the cellular bioenergetics. However, the results suggest that AgNPs interfere with the autophagy pathway, probably at the level of lysosomes, which may result in diminishing the efficacy of this protective mechanism (Fig. 9). Under our experimental conditions, defective autophagy flux is simultaneously being counteracted via the enhanced biodynamic of mitochondria as evidenced by the elongated structures of these organelles. Evidence for effective compensation in this manner is supported by the observation that physiological levels of ATP are maintained and AgNP-induced cellular death via the apoptotic pathway does not occur.

## Data Availability

Data are available from the authors.
